# Structural model and characteristics of kindergarten teachers’ occupational beliefs in China: A grounded theory approach

**DOI:** 10.3389/fpsyg.2022.976719

**Published:** 2022-11-07

**Authors:** Lei Zhang, Qiang Guo, Jie Zhu, Tingzhao Wang, Bisheng Hu

**Affiliations:** ^1^Changchun Normal College, Changchun, China; ^2^Hangzhou College of Preschool Education, Zhejiang Normal University, Hangzhou, China; ^3^Department of Special Education, Faculty of Education, Shaanxi Normal University, Xi'an, China; ^4^Zhejiang Bema Postdoctoral Workstation, Hangzhou, China

**Keywords:** kindergarten teachers, occupational beliefs, grounded theory, qualitative methods, characteristics of occupational beliefs

## Abstract

To explore the characteristics of kindergarten teachers’ occupational beliefs in China, 24 public and private kindergarten teachers were interviewed, their teaching was observed, and teaching reflection notes and educational essays were collected. A structural model was subsequently developed. Based on grounded theory, the information was coded at three levels and tested for theoretical saturation. The results demonstrated that the occupational beliefs of kindergarten teachers consisted of occupational cognition, occupational endurance, occupational expectation, and occupational intention, and these four core elements were not independent. Although there were characteristics common to public and private kindergarten teachers’ occupational beliefs, they also had distinct characteristics. The occupational beliefs of public kindergarten teachers were relatively stable, yet those of private kindergarten teachers were not. Moreover, most public kindergarten teachers were willing to stay in their teaching jobs. These findings reveal the composition and characteristics of Chinese kindergarten teachers’ occupational beliefs and the relationship between kindergarten teachers’ turnover and occupational beliefs.

## Introduction

As an important part of early childhood education in China, kindergartens primarily serve children aged 3–6. The quality of kindergartens plays an important role in the development of early childhood education. Factors such as staff and personal care routines are important indicators of kindergarten quality assessment (Li et al., 2016). Therefore, the quality and stability of teachers might directly affect the development of children in kindergartens (Jin, 2010; Chen, 2020). Teachers with professional knowledge, skills, and strong beliefs are highly accomplished in their work and tend to be happy (Wang M. et al., 2015). Studies have demonstrated that teachers’ professional identity and commitment affect their occupational well-being and the stability of the team of teachers (Wang et al., 2018). In particular, teachers’ occupational beliefs enhance teaching effectiveness and overcome burnout, perhaps becoming an important influencing factor in teacher development (Tang, 2010; Xiao, 2013). Thus, focusing on the intrinsic subjective attitudes of kindergarten teachers has become an important area of research in China. However, the occupational beliefs of early childhood teachers have not been widely examined. In addition to internal factors, the type of kindergarten (public program, private program), as an important external factor, significantly impacts teachers’ work attitude and teaching efficacy (Cao, 2010; Jia, 2017). It is of interest to determine whether the characteristics of teachers’ occupational beliefs differ between public and private kindergartens. Therefore, this study explored the occupational beliefs of public and private kindergarten teachers in China. Additionally, the present study helps construct a model of kindergarten teachers’ occupational beliefs that is consistent with China’s local context and clarifies the characteristics of public and private kindergarten teachers’ occupational beliefs. The findings can be used as a reference by educational administrations to formulate policies regarding kindergarten teachers’ occupational beliefs and can provide a basis for training programs related to teachers’ occupational beliefs.

## Literature review

### Early childhood education in China

Since 2010, early childhood education in China has entered a period called the great-leap-forward development (Pang et al., 2016). This has been driven by policies such as the “National Medium and Long-term Educational Reform and Development Plan (2010–2020).” Accordingly, the Ministries of Education and Finance and allied departments in other ministries are committed to providing financial and technical support for teacher training for early childhood education in China (Liang, 2019; Yuan and Yang, 2019). Kindergartens have also greatly expanded in numbers across China. By 2019, the proportion of children of appropriate age enrolled in kindergartens was 83.4%, with a further increase of 32.5% projected in the past decade (Yang and Yue, 2020). Despite the remarkable development of kindergartens in China, many shortcomings remain, such as the imbalance in the development of public and private kindergartens and a shortage of teachers. After the implementation of the “two-child” and “three-child” policies in China, these deficiencies became even more prominent (Hong and Jiang, 2018). Based on a gross enrollment proportion of 85%, a shortfall of at least 1.2 million teachers exists. In addition, teacher retention and turnover are serious challenges (Pang et al., 2016; Jiang and Shang, 2020). Owing to a deficiency of resources and low salaries, private kindergartens lose nearly one-third of teachers yearly (Feng et al., 2017), while a large proportion of teachers in public kindergartens experience burnout because of excessive workload (Li et al., 2019). Such kindergartens risk losing teachers who are willing to leave their jobs due to such burnout (Shang and Shen, 2017; Hong et al., 2021). Subsequently, some researchers have argued that retention of kindergarten teachers is related to internal and external conditions and the teachers’ own occupational beliefs (Tubbs and Dahl, 1991; Bernshausen and Cunningham, 2001; Mittapalli, 2008).

### Teachers’ occupational beliefs

Teachers’ occupational beliefs refer to teachers’ attitudes based on their knowledge of their profession regarding the value of their labor and professional development (Wang, 2000). Such attitudes involve teachers’ identification with their profession, love, evaluation, and recognition of the value of their labor (Shi and Zhang, 2010). Occupational beliefs enhance teacher efficacy, help maintain stability, and reduce the risk of resignation. Therefore, outlook and attitude are important factors in turnover in the teaching profession (Shi and Zhang, 2010; Quinn, 2011; Zhou, 2017). By contrast, Tang (2010) indicated that teachers’ occupational beliefs consist of a series of thoughts and value judgments regarding educational objectives, teaching processes, methods, teacher-student relationships, and self-development within the teaching profession. Some researchers have equated teachers’ personal beliefs with their occupational beliefs (Pajares, 1992; Lv, 2007; Chen, 2009). However, there is a clear difference between the two. Teachers’ personal beliefs refer to the stable and believed views teachers have developed during their lives and work. It has a broader scope, encompassing ideas and attitudes about nature, society, and education (Xiao, 2013). Teachers’ occupational beliefs are the viewpoints and attitudes about professional development that teachers develop in teaching and related matters, and they are relatively concentrated (Cui, 2012). Studies have shown that there are substantial differences between personal and occupational beliefs. Quinn (2011) explored the occupational beliefs of pre-service and in-service teachers through a confirmatory factor analysis. The results indicated that teachers’ occupational beliefs included seven core elements: professional development, occupational despair, desired collaboration with colleagues, commitment to the profession, unions, control over instruction, and instructional preparedness. Influenced by collectivism and knowledge-oriented traditions, Chinese kindergartens focus on group instruction in children’s education, emphasizing educational discipline and academic knowledge. Children’s interests and educational needs received less attention (Li et al., 2016). Although kindergartens in China, similar to those in the United States, are divided into public and private kindergartens, most Chinese private kindergartens are poorly funded and equipped. These private kindergartens serve children from low-and middle-income families (Hu and Li, 2012). As Quinn’s study was grounded in the US cultural background, its findings need to be further tested outside that environment to determine their consistency and application. In addition, few studies have assessed the structure of Chinese kindergarten teachers’ occupational beliefs. Subsequently, it is unclear whether the findings mentioned above apply to the occupational beliefs of Chinese kindergarten teachers.

### Occupational beliefs of Chinese teachers

With the continuous development of education in China, researchers have explored teachers’ occupational beliefs through theoretical and empirical approaches. Wang (2000) proposed the concept of Chinese teachers’ occupational beliefs and argued that teachers’ perceptions and needs are the two most influential factors. Luo (1987) explored the formation process of young teachers’ attitudes and beliefs. The study found that teachers’ occupational beliefs changed with increasing seniority. Occupational beliefs also exhibit diversity among career stages. For example, pre-service teachers are generally open-minded and positive (Zhao and Zuo, 2016). By contrast, there were differences in the occupational beliefs of primary and secondary school teachers in Northwest China concerning seniority, diploma, and professional title (Du, 2018). Based on the 2015 Program for International Student Assessment data on science teachers, Liu et al. (2019) compared the occupational beliefs of teachers from China and the United States. The results demonstrated that the occupational beliefs’ scores of Chinese teachers were lower than those of teachers in the US. The occupational beliefs of teachers in colleges and universities varied greatly among regions, and those of young teachers recruited to colleges or universities in the southeastern coastal cities of China were generally not firm (Wang, 1999). These teachers were concerned about external working conditions, believed that the teaching profession had low prestige, and rated their expectations of teaching as low. Some teachers indicated that they would give up their current jobs if more generously paid positions were available. Moreover, teachers in these locations reported low satisfaction with their salary, low role expectations, and low satisfaction with promotion opportunities and the career environment (Wu and Luo, 2015). However, the occupational beliefs of college teachers in the Beijing–Tianjin–Hebei region were relatively positive (Yao, 2021). Although scholars have discussed the issue of teachers’ occupational beliefs in China, there is a relative lack of research on kindergarten teachers’ occupational beliefs. Consequently, the characteristics of such occupational beliefs, therefore, need further research.

### The impact of kindergarten’s type (public/private program) on teachers

Currently, there are significant differences between public and private kindergartens in China. Public kindergartens are typically better than private kindergartens in terms of management and salaries in China (Hong and Hua, 2015). Most public kindergarten teachers have establishment, and their employment is more stable than in private kindergartens (Jia, 2017; Qiu et al., 2020). The job descriptions and responsibilities of teachers in public kindergartens are less extensive than those of teachers in private kindergartens, and the working hours of the former are shorter (Chiu and Wei, 2014). These factors lead to differences in work-related cognition and work values between public and private kindergarten teachers (Zhang et al., 2014). The professional identity of public kindergarten teachers in China is stronger than that of private kindergarten teachers. Public kindergarten teachers’ professional self-knowledge and motivation are also significantly higher than those of private kindergarten teachers (Jia, 2017). Further, Xu et al. (2017) found that the level of public kindergarten teachers’ professional commitment is higher than that of private kindergarten teachers. Moreover, some studies have demonstrated that teachers in public kindergartens score higher on developmentally appropriate practice beliefs than teachers in private kindergartens (Liu, 2007). Hence, the question arises as to whether the type of kindergarten in China affects teachers’ occupational beliefs. It is important to elucidate the characteristics of public and private kindergarten teachers’ occupational beliefs; this has rarely been explored.

### The present study

Based on the relevant research, the present study focused on the occupational beliefs of kindergarten teachers in China. According to grounded theory methods, this study utilized bottom-up qualitative analysis to explore the structure of occupational beliefs among kindergarten teachers in China. The study also analyzed and explored the characteristics of occupational beliefs among teachers in public and private kindergartens. It is anticipated that these findings will provide a reference for the core elements and characteristics of the occupational beliefs of kindergarten teachers in China.

## Research design

### Research methods and tools

This study adopted a qualitative research approach. The grounded theory is a theory that enables continuous induction and comparative analysis of raw data to obtain theories (Cutcliffe, 2000; Chen, 2015). The grounded theory has applicability exploring complex logical relationships and factors (Han and Zhou, 2011; Chen, 2022). There are several reasons for this study to adopt the grounded theory method. First, the grounded theory focuses on collecting data openly and generating theories from primary data, which is convenient for extracting the core elements and constructing models of kindergarten teachers’ occupational beliefs. Second, the grounded theory method has a strict coding process, which continuously compares and analyzes on the basis of primary data and coding at different levels. This enables the obtained elements and model of kindergarten teachers’ occupational beliefs to have strong rigor. Finally, the grounded theory is based on phenomenal information and empirical summaries, which have strong guiding value for practice. Based on grounded theory, interviews were conducted in the natural teaching setting. We obtained teaching notes and other raw materials and extracted the main factors associated with kindergarten teachers’ occupational beliefs through initial and axial coding. Then, selective coding was used to explore the logical relationship among the factors and to construct a theoretical model of kindergarten teachers’ occupational beliefs.

NVivo qualitative analysis software can process text, interviews, pictures, audio, and video data to help researchers determine the logical relationships among concepts through decision tree nodes or free nodes (Lakeman, 2009). This study used NVivo11 Plus qualitative analysis software to encode and analyze the original data, combined with the grounded theory, to reveal the content structure of kindergarten teachers’ occupational beliefs.

### Participants

In China, kindergartens can be typically divided into public and private kindergartens (Qin et al., 2011). For the purpose of the current study, one presentative public kindergarten and one private kindergarten in Changchun city, located in Northeast China, were selected by a team of early childhood education experts based on the mainstream classification of kindergartens in China and indicators of kindergarten quality assessment (e.g., material conditions, teacher quality, teacher-child interaction, curriculum, parent community involvement, and child development) (Ma et al., 2019). The public kindergarten is a provincial demonstration kindergarten^1^. Additionally, the private kindergarten matched the public kindergarten in terms of school size, facilities, and student-teacher ratio, ensuring the equivalence of the selected kindergartens in their basic characteristics. Next, four teachers from each of the three age groups (K1: 3–4, K2: 4–5, K3: 5–6) were selected from each of the participating kindergartens with consideration of the teachers’ years of teaching experience. Finally, 12 public kindergarten teachers and 12 private kindergarten teachers were selected. Their basic information is provided in Table 1. Theoretical sampling principles were followed throughout the data collection and analysis to reach theoretical saturation, thus avoiding new concepts and categories (Corbin and Strauss, 1990).

**Table 1:** Basic participant information.

Number	Age	Class^a^	Seniority	Educational level	Type of kindergarten
M1	36	K3	16	Postgraduate	Public
M2	36	K3	15	Postgraduate	Public
M3	25	K3	2	Undergraduate	Public
M4	24	K3	1	Undergraduate	Public
M5	28	K2	5	Undergraduate	Public
M6	28	K2	4	Postgraduate	Public
M7	28	K2	6	Junior college	Public
M8	34	K2	10	Undergraduate	Public
M9	35	K1	11	Undergraduate	Public
M10	28	K1	8	Junior college	Public
M11	28	K1	5	Undergraduate	Public
M12	26	K1	6	Junior college	Public
M13	33	K3	10	Junior college	Private
M14	30	K3	8	Junior college	Private
M15	22	K3	2	Junior college	Private
M16	28	K3	6	Junior college	Private
M17	27	K2	3	Undergraduate	Private
M18	28	K2	5	Undergraduate	Private
M19	24	K2	3	Junior college	Private
M20	23	K2	1	Undergraduate	Private
M21	35	K1	12	Junior college	Private
M22	28	K1	4	Undergraduate	Private
M23	24	K1	4	Junior college	Private
M24	21	K1	1	Junior college	Private

a Kindergartens (3–6 years old) in the Chinese Mainland are divided into K1 (3–4 years old), K2 (4–5 years old), and K3 (5–6 years old) classes. In this study, the teachers were selected according to these stages.

### Data collation

Original data on kindergarten teachers’ occupational beliefs were collected in Chinese environment. The information was organized and coded, primarily through interviews, observation of teachers at work, and essays and reflection data, and then translated into English in the data analysis. Data reliability and validity were ensured through the triangulation of these data sources. We received ethical approval for the study from the Ethics Committee of [the second author’s university]. The first author explained the purpose and the content of the study to the principals of the two kindergartens and obtained their approval. The researchers then recruited participants from two kindergartens. Prior to data collection, kindergarten teachers who volunteered to participate in the study were informed of the purpose of the study and privacy protections, and their informed consent was obtained. Interview data were collected first, followed by observational and reflective data. Data collection and analysis were conducted simultaneously based on the principles of the grounded theory data analysis. All interview data were coded and organized after each interview and compared with the previous interviews’ data until no new concepts or categories were generated. Observational and reflection data were collected in the same manner.

#### Interview data

Based on the literature analysis and purpose of this study, a preliminary interview protocol was developed (Wang, 2000; Zhou, 2016). To ensure that the interview protocol was effective in collecting information, three early childhood education experts were invited to revise the interview protocol. The revised interview protocol was reviewed and modified by two kindergarten teachers from public and private kindergartens who did not participate in the formal interviews. They both had more than 5 years of experience and were familiar with kindergarten teaching. The modification of the interview protocol was based on whether the language was easy for kindergarten teachers to understand. The interview protocol was finalized after all ambiguity had been removed.

One-on-one semi-structured interviews were conducted with the 24 kindergarten teachers for 30 to 40 min, in the conference rooms of the two kindergartens. One researcher conducted the interviews and recorded them using a tape recorder, with the interviewees’ permission. The interviewees were the 24 teachers who participated in this study. The purpose of the study and the connotations of occupational beliefs were explained before the formal interviews, so that interviewees had a preliminary understanding of the content related to occupational beliefs. A three-level interview strategy was used (Wang and Tian, 2022). The first level is the “situational introduction” (e.g., Do you think kindergarten teachers have interesting jobs?), the second level was the “core interview” (e.g., What difficulties have you encountered in teaching? How did you deal with these problems?), and the third level was the “in-depth interview” (e.g., What do you think there are any other manifestations of good occupational beliefs of kindergarten teachers?). The researcher followed up with meaningful responses at appropriate times during the interview.

#### Observational data

The observational data primarily consisted of observations of the kindergarten teachers’ and children’s activities. Teachers’ teaching and daily activities with children were videotaped using cameras. One observation was recorded per school day (8 hours), and the research team determined the criteria for transcribing the observational data. The transcription criteria focused on the following areas: teachers’ attention to children; teachers’ interactions with children; children’s affectionate behavior toward teachers; teachers’ relationships with colleagues, children” parents, and leaders; teachers’ work environment; and kindergarten rules and regulations. Video materials were transcribed into text based on the transcription criteria by two researchers. Using a stratified sampling of small, medium, and large classes, two teachers were selected from each class for on-site observation of teachers at work and teachers’ records. One researcher obtained six records.

#### Essay and reflection data

Teaching reflection notes and educational essays were obtained with the consent of the teachers involved in the study. These essays and reflections focused on teachers’ daily teaching activities, teaching improvement, and teachers’ self-reflection. They were written for teachers to improve their teaching skills, academic abilities, or complete training assignments. A total of 24 teaching reflection notes and 12 educational essays were obtained.

## Data analysis

The audio recordings of the interviews were converted into textual materials and imported into NVivo11 Plus for processing and analysis alongside observation notes, teaching reflection notes, and educational essays. The data were encoded consecutively by two independent researchers. The two researchers were proficient in the grounded theory coding method. The research team discussed and determined the coding criteria before formal coding, and the two researchers then coded independently according to the coding criteria. More than 10% of the transcripts were sampled for coding similarity comparison. The overall inter-rater agreement, which was 80% or above, indicates that the coding procedure was reasonably reliable. After the coding was complete, the coding nodes were checked, and nodes with consistent coding results were retained. A third researcher encoded the inconsistent parts of the material to ensure coding reliability (Yao and Jin, 2009). Following the grounded theory methods, the collected materials were coded in three stages: open coding, axial coding, and selective coding (Strauss and Corbin, 1998).

### Initial coding

The original statements were coded under the theme of “kindergarten teachers’ occupational beliefs.” To ensure the original semantics of the data were preserved, the initial concepts were coded using concepts or words from the original statements. After several comparisons and mergers, 707 nodes were obtained; 24 original concepts and 11 initial categories were extracted by conceptualizing and organizing the original data (Table 2).

**Table 2:** Initial coding of the structure of kindergarten teachers’ occupational beliefs.

Initial category	Original concept	Source of materials	Nodes	Original statements
Understanding of occupational attributes	Time load	28	44	The pace of kindergarten work is fast, with daily activities and teaching competition taking up all the time. It is normal to work overtime to prepare lessons and the classroom.
	Trivial content	38	62	The teacher’s work is very hard and trivial, and the children’s eating, drinking, and sleeping are all our responsibility.
	Love and responsibility	20	24	Early childhood teachers are professionals who work with young children who do not yet have the ability to take care of themselves and judge things, so teachers need to be caring, attentive, and responsible.
Understanding of professionalism	Educational knowledge and ability	28	36	Being a kindergarten teacher is not as simple as playing with children but also requires a lot of professional knowledge and abilities.
	Irreplaceable professional	10	12	Not just anyone can be a kindergarten teacher. Teachers must be professionally qualified and licensed.
Understanding of occupational value	Social value	16	24	The occupation of a kindergarten teacher is of great value to the growth of young children and the development of preschool education.
	Personal value	18	20	I like using my own educational experience to help children to grow and improve. It gives me a great sense of accomplishment and makes me feel that my work is worthwhile.
Occupational security expectation	System guarantee	18	36	I hope that the kindergarten management will become more systematic. If the leaders pay more attention and provide more support to the teachers, the teachers will be more motivated to work.
	Economic security	30	56	Compared with primary and secondary school teachers, kindergarten teachers’ salary is generally low.
Occupational development expectation	Professional development	12	20	We hope kindergartens can provide diversified development platforms and learning opportunities, such as training and learning, promotion opportunities, and practical teaching competitions.
	Self-enhancement	10	20	I hope to improve my abilities at work by, for example, the accumulation of teaching experience, education improvement, and communication skills.
Occupational prestige expectation	Social status	12	14	Although the country began to pay attention to preschool education, the social status of preschool teachers is still not high, I hope the social status of preschool teachers can be improved.
	Recognition from others	20	30	I hope to get recognition, support, and cooperation from leaders, parents, and children.
Tolerance	Emotional control	24	54	In addition to heavy tasks, it is necessary to deal with various communication problems between children and parents. I must have better emotional control skills and maintain my peace of mind.
	Behavioral control	10	16	In an unexpected situation, we can maintain our sanity and control our behavior to ensure that teaching activities are carried out properly.
Resolving power	Positive coping	18	28	When dealing with “problem children,” I need to actively adjust my mind, look for the cause, and try to solve it; when I encounter problems that cannot be solved, I will consult a more experienced teacher.
	Get out of trouble	9	14	When I encounter teaching problems, I can effectively solve them using my accumulated experience or discussing them in teaching and research activities.
Teaching willingness	Staying intention	24	30	I will continue to work in this occupation; this is my career.
	Leaving intention	10	15	If there is a better choice, I might leave the occupation at any time.
Emotional willingness	Work well-being	30	50	After being with the children every day, the teachers feel a sense of happiness and achievement. So, this is a happy occupation.
	Interest preference	20	38	I like children, am willing to be with children, and am good at communicating with children. So, I like this occupation very much.
	Work enthusiasm	18	22	Although the kindergarten teachers are very busy, most teachers are willing to do it, and everyone is full of enthusiasm and motivation.
Responsibility willingness	Responsibility	16	28	Sticking to this occupation is not only because of love but also a kind of responsibility. While completing the tasks, you must have goals and plans to do the job well.
	Perfunctory	12	14	In addition to work, I must take care of my family, so I do not have very much energy and time to prepare lessons well every time.

#### Axial coding

Based on the original concepts and categories, axial coding further explores and represents the relationships among the categories (Strauss and Corbin, 1998). Based on the 24 initial categories, this study further distinguished the connotations and extensions of the concepts and investigated their relationship. Finally, the kindergarten teachers’ occupational beliefs were obtained in four main categories of occupational cognition, occupational endurance, occupation expectation, and occupational intention (Table 3).

**Table 3:** Kindergarten teachers’ occupational beliefs spindle coding analysis table.

Main category	Initial category	Relationship connotation
Occupational cognition	Understanding of occupational attributes	Kindergarten teachers’ understanding of occupational attributes, such as working hours, intensity, objects, and content, is the basis of occupational cognition.
	Understanding of professionalism	Kindergarten teachers’ knowledge and competence in education and professionalism are prerequisites for forming a scientific occupational understanding.
	Understanding of occupational value	Kindergarten teachers’ understanding of the social and personal value of their occupation can promote the formation of their own labor value identity.
Occupational expectation	Occupational security expectation	The expectation of kindergarten teachers for material and spiritual rewards of the occupation enhances their intention to remain in the profession.
	Occupational development expectation	The expectation of kindergarten teachers for occupational development and self-improvement is the pursuit of self-worth.
	Occupational prestige expectation	Kindergarten teachers’ expectations of positive social status and recognition by others are an affirmation of their occupational value.
Occupational endurance	Resistance	Kindergarten teachers’ tolerance for frustration and stress enables them to ensure a calm state of mind and normal teaching behavior.
	Resolving power	Kindergarten teachers’ ability to resolve frustration and stress enables teachers to actively cope with and overcome difficulties.
Occupational intention	Teaching willingness	A firm willingness to teach is the fundamental motivation for kindergarten teachers to stay in their jobs.
	Emotional willingness	The emotional support for continuing to be teachers derives from kindergarten teachers’ love of the job, enthusiasm, and happiness.
	Responsibility willingness	Kindergarten teachers’ sense of responsibility for their occupation is an inherent requirement for doing their job well.

### Selective coding

Through a systematic analysis, selective coding obtains a “core category” from the existing conceptual categories and subcategories, which establishes a relationship between the main category and the core categories, thus constructing a complete theoretical model (Pepe et al., 2017). Based on the initial and axial coding, this study generated a highly abstract generalization, clarified the inherent logical structure of its theory, and refined the core category of kindergarten teachers’ occupational beliefs. The relationships among the core and main categories were systematically analyzed, and a theoretical model of kindergarten teachers’ occupational beliefs was constructed (Table 4).

**Table 4:** Typical relationship structure and connotation of main categories.

Typical relationship structure	Relationship structure connotation
Occupational cognition—Kindergarten teachers’ occupational beliefs	Cognition is the basis for beliefs; scientific occupational understanding is conducive to forming firm occupational beliefs among kindergarten teachers.
Occupational endurance—Kindergarten teachers’ occupational beliefs	Occupational endurance refers to the ability of kindergarten teachers to tolerate and resolve frustration. The stronger the occupational endurance of kindergarten teachers, the stronger their occupational beliefs.
Occupational expectation—Kindergarten teachers’ occupational beliefs	Occupational expectations are the aspiration and pursuit of opportunities within an occupation, reflecting attitudes and beliefs toward future careers, and are also the internal motivation for forming kindergarten teachers’ occupational beliefs.
Occupational intention—Kindergarten teachers’ occupational beliefs	Whether kindergarten teachers enjoy and are willing to engage in their occupation are important manifestations of occupational beliefs. The stronger the occupational will, the stronger the occupational belief.

### Verification of theoretical saturation

To ascertain the validity of the model of kindergarten teachers’ occupational beliefs, this study tested the theoretical saturation of the four interview materials, four sets of teaching reflection notes, two educational essays, and two teaching observation records. The coding test process found no new coding nodes, initial concepts, or main categories. Therefore, it was considered that the model of kindergarten teachers’ occupational beliefs constructed in this study reached theoretical saturation.

## Results

### Model of the occupational beliefs of kindergarten teachers and structural relationships

**Figure 1:**
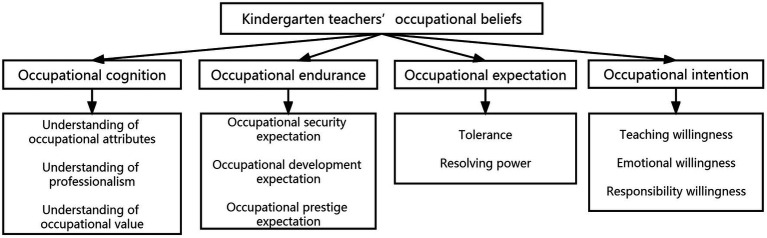
Model of kindergarten teachers’ occupational beliefs.

After systematic coding and the grounded theory analysis, as illustrated in Figure 1, a model of kindergarten teachers’ occupational beliefs was constructed consisting of four main categories (i.e., occupational cognition, occupational endurance, occupational expectation, and occupational intention) and 11 subcategories. The meaning and content of these elements are described in Tables 3, 4 above.

**Figure 2:**
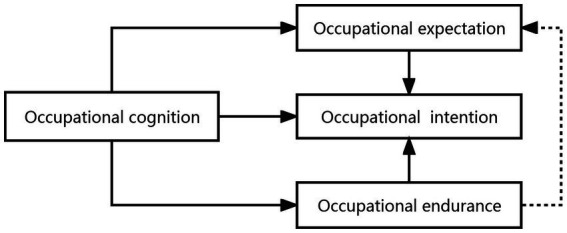
Structural relationship of kindergarten teachers’ occupational beliefs.

As illustrated in Figure 2, the model illustrates the relationship between the elements of kindergarten teachers’ occupational beliefs. Occupational cognition is the basis of kindergarten teachers’ occupational beliefs, which are produced in the process of cognition. Occupational cognition directly promoted the formation of kindergarten teachers’ occupational beliefs and affected the formation of their occupational endurance, expectation, and intention. Occupational endurance was an important factor in the strength of kindergarten teachers’ occupational beliefs. The interviews revealed that the stronger the occupational endurance, the stronger the kindergarten teachers’ occupational intention. This, in turn, promoted the strength of kindergarten teachers’ occupational beliefs.

Occupational expectation was the internal force driving the formation of kindergarten teachers’ occupational beliefs. The higher the occupational expectation, the stronger the kindergarten teachers’ willingness to engage in the occupation, thus stimulating their occupational beliefs.

Occupational intentions were the fundamental embodiment of kindergarten teachers’ occupational beliefs, and occupational intentions were influenced by occupational cognition, expectation, and endurance. It has been demonstrated that the teachers’ resilience positively influences their occupational expectations (He et al., 2019), and the model also demonstrates that teachers’ endurance influences occupational expectations; however, this needs further validation.

### Discovery of occupational beliefs of kindergarten teachers

**Figure 3:**
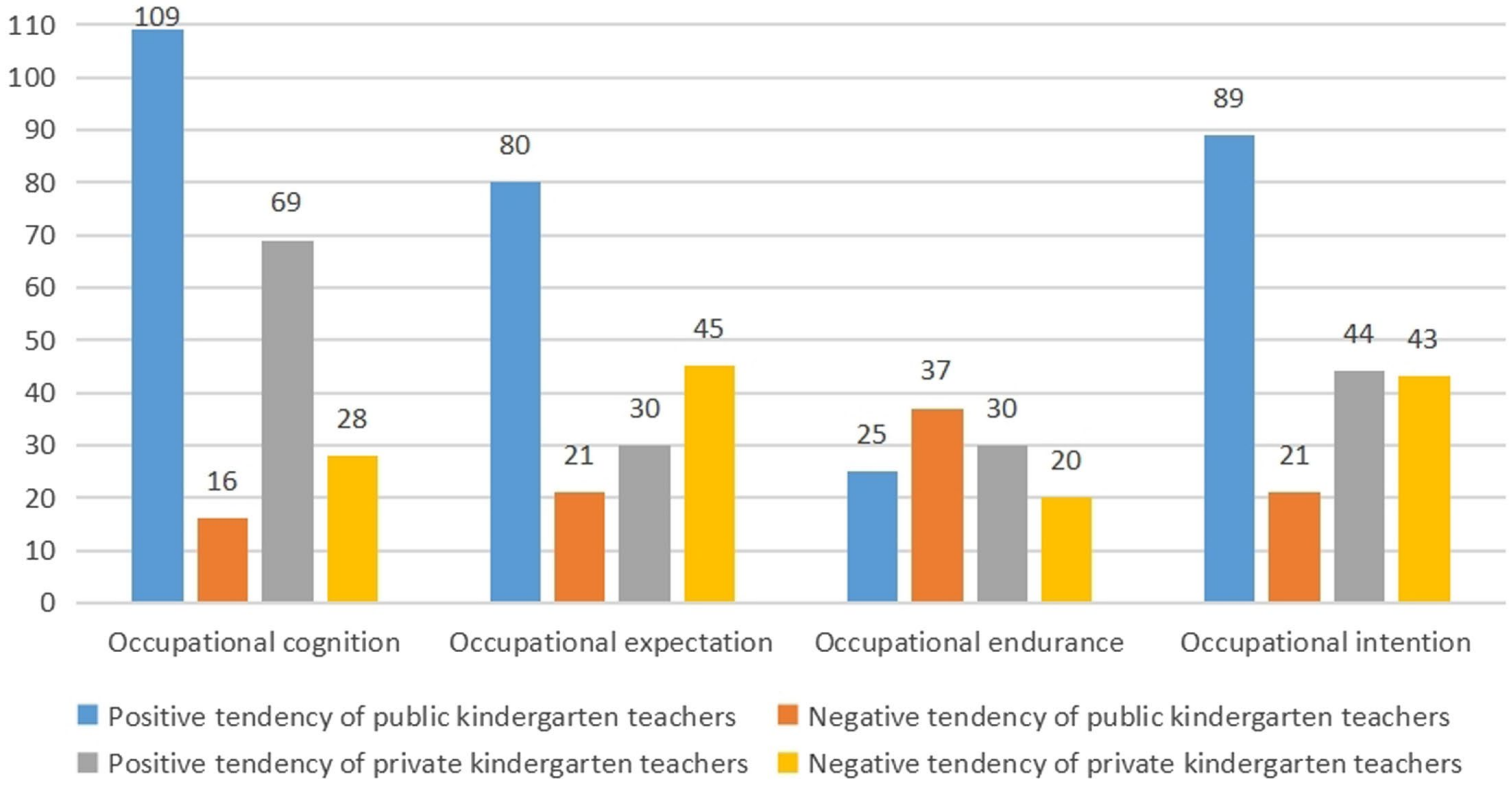
Distribution of the number of initial coding nodes of positive and negative tendencies of teachers on the four elements in public and private kindergartens.

Kindergarten teachers’ occupational beliefs comprised occupational cognition, endurance, expectation, and intention. These four elements were not isolated, but rather integrated. Although the teachers in public and private kindergartens had some common attributes in their occupational beliefs during the interviews, notable differences were found between the two types of kindergarten teachers in these four elements, as illustrated in Figure 3. Some initial coding nodes expressed positive tendencies, whereas others expressed negative tendencies. The ratios of the number of initial coding nodes of positive and negative tendencies among the four elements of occupational beliefs were used to further reveal the characteristics of public and private kindergarten teachers’ occupational beliefs. The results can be summarized as follows.

### The occupational cognition was positive among public and private kindergarten teachers

Occupational cognition refers to kindergarten teachers’ awareness of the attributes, professionalism, and value of their occupation. The number of initial coding nodes for occupational cognition was 222, among which the proportion of the positive cognitive nodes of the public kindergarten teachers was the largest (*n* = 109, 49.10%), followed by private kindergarten teachers’ positive cognitive nodes (*n* = 69, 31.08%), private kindergarten teachers’ negative nodes (*n* = 28, 12.61%), and public kindergarten teachers’ negative nodes (*n* = 16, 7.21%). This indicates that most teachers in public and private kindergartens have positive perceptions in terms of their occupational cognition. Kindergarten teachers might have a relative understanding of their professional attributes and be aware of the value of their work.

### The occupational expectation of public kindergarten teachers was positive, while that of private kindergarten teachers was negative

Occupational expectation refers to teachers’ aspirations and pursuit of their career and their attitudes toward career development. Public teachers had 176 initial coding nodes of occupational expectation, with a greater proportion of positive expectation nodes (*n* = 80, 45.45%) and a smaller proportion of negative expectation nodes (*n* = 21, 11.93%). In contrast, private teachers had a small number of positive expectation nodes (*n* = 30, 17.05%) and a large number of negative expectation nodes (*n* = 45, 25.57%). This indicates that teachers in public kindergartens have higher career development expectations and are more excited about their future development. Private teachers are relatively pessimistic in terms of career development.

### Public kindergarten teachers were more negative in terms of occupational endurance than private kindergarten teachers

Occupational endurance refers to the kindergarten teachers’ ability to tolerate and resolve frustration. There were 112 nodes in the initial code of occupational endurance, with small and large proportions of positive nodes among public kindergarten teachers (*n* = 25, 22.32%) and private kindergarten teachers (*n* = 30, 26.79%), respectively. However, in the aspect of the negative nodes of occupational endurance, the node proportion of public kindergarten teachers was larger (*n* = 37, 33.04%), whereas that of the private kindergarten teachers was smaller (*n* = 20, 17.86%). This demonstrates that public kindergarten teachers are relatively passive in their occupational endurance. However, private kindergarten teachers were more optimistic about the difficulties and could easily endure and relieve stress.

### Occupational intention was strong among public kindergarten teachers and weak among private kindergarten teachers

Occupational intention refers to the kindergarten teachers’ enjoyment of the occupation and their attitude toward long-term engagement in the occupation. There were 197 initial coding nodes for occupational intention. Public kindergarten teachers had a larger proportion of positive nodes (*n* = 89, 45.18%) and a smaller proportion of negative nodes (*n* = 21, 10.66%). The proportions of positive nodes (*n* = 44, 22.34%) and negative nodes (*n* = 43, 21.83%) were similar for private kindergarten teachers. This indicates that public kindergarten teachers might have strong occupational intentions, and private kindergarten teachers have weaker occupational intentions.

### Realistic patterns of kindergarten teachers’ occupational beliefs

Analysis of the above four core elements’ coding characteristics revealed that teachers’ occupational beliefs in public and private kindergartens had different but realistic patterns.

#### Public kindergarten: “Duty” in stability

The occupational beliefs of teachers in the public kindergarten were relatively stable. Although most public kindergarten teachers had stable posts, there remained hidden attrition. In the interviews, more than 90% of the public kindergarten teachers thought their work was well-organized, and their income was stable, representing a relatively satisfactory situation. Seventy-five percent of the teachers felt that they had development opportunities, a sense of achievement in their work, and self-worth. Although many public kindergarten teachers felt that they should perform well after choosing this occupation, most indicated that they were working under great pressure. Some teachers experienced job burnout.

#### Private kindergarten: Characterized by frustration

The occupational beliefs of teachers in the private kindergartens were unsound. The turnover of teachers in the private kindergarten was high, and some in-service teachers considered leaving. In the interviews, more than 85% of the private kindergarten teachers complained that their workload was not proportional to their income and that their career development was not guaranteed. More than half of the teachers said that kindergarten administrators did not understand their work or any suggestions they made. Furthermore, these teachers thought some parents regarded them as “omnipotent nannies.” Given the dissatisfaction with salaries and working conditions, some private kindergarten teachers said they would prefer an amount of “work” consistent with their employment status, and a considerable number of private kindergarten teachers said they had thought of leaving or planned to leave their current job.

## Discussion

Based on grounded theory, this study explored a model of kindergarten teachers’ occupational beliefs and investigated these beliefs concerning a matrix of four core elements: Occupational cognition was the basis of occupational beliefs, occupational endurance was an important factor in occupational beliefs, occupational expectations were the internal driving force in the formation of occupational beliefs, and occupational intentions were the fundamental embodiment of the formation of occupational beliefs. The occupational beliefs among teachers in public and private kindergartens had different characteristics. Overall, the occupational beliefs among teachers in public kindergartens were relatively positive and stable, while those in private kindergartens were relatively negative and unstable.

Through the coding analysis and inductive generalization, the current study obtained four core elements of kindergarten teachers’ occupational beliefs: occupational cognition, occupational endurance, occupational expectation, and occupational intention. This result is consistent with the dimensions of teachers’ occupational beliefs extracted by Quinn (2011). Most teachers were aware of the important role of their educator-related knowledge and skills. Teachers with different occupational beliefs differed in their emotional response to teaching, the role of responsibility, coping mechanisms for stress, and attitudes toward career development. Unlike Quinn’s (2011) study, the current study did not extract information on *gonghui* (similar to the concept of “teachers’ unions” in a western context). Diverse cultural backgrounds may influence such unions. Although there are *gonghui* in kindergartens in China, these did not play a practical support role in the teachers’ activities. Accordingly, they received relatively little attention from the teachers. By contrast, He (2013) proposed a binary structure framework in which teachers’ competence and professional preferences were considered the core elements of occupational beliefs. The model of kindergarten teachers’ occupational beliefs in this study not only focused on teachers’ cognition and competencies but also considered teachers’ expectations and intentions when engaging in their careers. Thus, the current model better reflects kindergarten teachers’ occupational beliefs.

Although some researchers have equated teachers’ beliefs with their occupational beliefs (Basckin et al., 2021), the two differ in connotation and extension (Chen, 2009; Cui, 2012). Some researchers have considered teacher beliefs as an internal perspective or attitude informed by nature, society, and education (Zhao, 2004). Its extension is relatively comprehensive, including life, educational, and occupational beliefs (Chen, 2009). At the same time, teachers’ occupational beliefs emphasize teachers’ attitudes toward their occupation. This aspect involves their identification with, positive evaluation of, and perception of the occupation (Wang, 2000). In this study, kindergarten teachers’ occupational beliefs were primarily reflected in four aspects: occupational cognition, occupational endurance, occupational expectation, and occupational intention. These findings further clarify the structural content of kindergarten teachers’ occupational beliefs and help distinguish their beliefs concerning their core elements.

The constituent elements of occupational beliefs are important for kindergarten teachers, while their influences and manifestations vary. The elements are independent of and influence each other. In kindergarten teachers’ teaching activities, a good occupational cognition is the basis for the emergence of occupational beliefs. This awareness enables teachers to solve difficult problems in their work, thus enhancing their professional endurance. This further results in teachers demonstrating a strong willingness to grow professionally as well as a high level of expectation for professional development. During the interviews, several teachers noted that “overcoming difficulties and the sense of accomplishment in helping young children grow strengthened their confidence in working.” Overall, teachers’ professional beliefs have a holistic effect that cannot be replaced by individual elements. Teachers who have strong and scientific occupational beliefs are fully engaged in education and are motivated, proactive, and creative. This has an important impact on stabilizing the teaching force and improving the quality of education (Luo, 1987; Wang, 2000).

The study’s results demonstrated that teachers in public kindergartens were more positive than those in private kindergartens in terms of occupational cognition, expectation, and intention. Surprisingly, public teachers were relatively weak in this respect. Teachers in private kindergartens were strong in terms of occupational endurance, which represents an interesting phenomenon. Public teachers were generally better equipped with resources, and private teachers were at a disadvantage in this respect. This contrast between the groups of teachers might be associated with factors such as the stress faced by both categories of teachers (Qiu et al., 2020). Teachers in public kindergartens were required to teach, conduct scientific research, and participate in various competitions. In addition, they faced pressure to obtain professional titles. These job characteristics placed a high demand on teachers’ occupational endurance. Although teachers could withstand and cope with some of their task-related demands, they also exhibited a sense of helplessness when faced with multiple challenges. Conversely, the private kindergarten teachers had fewer sources of stress. They were under no pressure to obtain higher professional qualifications and saw no need to engage in scientific research. Therefore, the private kindergarten teachers worked under less pressure and had fewer demands on their occupational endurance, and the private kindergarten teachers were more able to cope with the stressors they experienced.

The occupational beliefs of public kindergarten teachers were relatively stable, while those of private kindergarten teachers were somewhat unstable. There may be several reasons for this finding. First, the organization type is an important factor that could affect the stability of teachers’ occupational beliefs. Public kindergartens are funded by the government and managed by government departments, such as the Bureau of Education. Teachers often have occupational security, with high income and management structure guarantees. Private kindergartens are privately funded and managed. Their salary and training opportunities are not guaranteed (Wang M. et al., 2015). Thus, there is an increased likelihood that teachers will leave (Yu and Gao, 2019; Qiu et al., 2020). Second, social recognition also affects teachers’ occupational beliefs. Teachers in public kindergartens are excellent professionals, as they are selected through professional examinations. The facilities and teaching environment in public kindergartens are also better than those in private kindergartens. Therefore, the public has a high degree of respect for public kindergarten teachers. In the case of private kindergartens, both the quality of teachers and facilities are relatively poor, which leads to relatively low public regard for private kindergarten teachers (Teng and Jiang, 2018; Wu, 2021). In addition, teachers’ cognition regarding occupation and self-realization are also important factors underlying differences in occupational beliefs (Qin et al., 2011). Public kindergarten teachers have received at least 4 years of undergraduate education and have relatively superior teaching resources. Thus, they can achieve greater achievement from the teaching process. Private kindergarten teachers have fewer opportunities for self-presentation, and their self-realization is relatively low (Zheng et al., 2021).

## Conclusion

This study conducted an exploratory analysis of the dimensions that describe occupational beliefs among kindergarten teachers using grounded theory. The data yielded realistic and reliable conclusions. However, there were the following shortcomings. First, this study overcame biases and preconceptions in coding using grounded theory and the software NVivo11 Plus. However, the construction and coding of the theoretical model remains subjective and could be flawed owing to the involvement of the researchers. Future research could consider adding coders to obtain a more reliable theoretical model of kindergarten teachers’ occupational beliefs. Second, owing to the small sample size of this study, the results need to be further verified. Future research could expand the sample size and develop a scale based on the dimensions of the theoretical model constructed in this study. Quantitative analyses with large-sample data could be conducted to obtain more robust conclusions. Finally, although this study explored a model of kindergarten teachers’ occupational beliefs and the relationship among the model elements in China, which provided a new perspective on kindergarten teachers’ occupational development, it could not reveal the influence of other factors on the model. Thus, subsequent research could further explore the occupational beliefs of kindergarten teachers from the perspective of other possible influential factors.

## Data availability statement

The raw data supporting the conclusions of this article will be made available by the authors, without undue reservation.

## Ethics statement

The studies involving human participants were reviewed and approved by Human Research Ethics Committee of Zhejiang Normal University. The patients/participants provided their written informed consent to participate in this study.

## Author contributions

QG and LZ designed this study, collected the data, and wrote and revised the manuscript. JZ wrote and revised the manuscript. TW and BH revised the manuscript. All authors contributed to the article and approved the submitted version.

## Funding

This work was supported by the National Social Science Fund of China (Grant no. 21&ZD293 to TW), the Social Science Fund of Shaanxi in China (Grant no. 2022P025 to TW), the Key Project of Changchun Education Science in 2021(Grant no. JKBLX2021024 to LZ), and the Social Science Fund of Xi’ an in China (Grant no. 22JY22 to TW).

## Conflict of interest

BH was employed by Zhejiang Bema Postdoctoral Workstation.

The remaining authors declare that the research was conducted in the absence of any commercial or financial relationships that could be construed as a potential conflict of interest.

## Publisher’s note

All claims expressed in this article are solely those of the authors and do not necessarily represent those of their affiliated organizations, or those of the publisher, the editors and the reviewers. Any product that may be evaluated in this article, or claim that may be made by its manufacturer, is not guaranteed or endorsed by the publisher.

## Footnotes

^1^Provincial demonstration kindergartens are top-level kindergartens that play a leading role in fully implementing educational policy, exploring the laws of education, and summarizing and promoting experience. They are tasked with mentoring general kindergartens. The kindergartens are evaluated and recognized by the Jilin Provincial Education Administration, based on the relevant Chinese documents, and on the criteria for model kindergartens, formulated in accordance with the local circumstances.
